# Plumbagin rescues the granulosa cell’s pyroptosis by reducing WTAP-mediated N6-methylation in polycystic ovary syndrome

**DOI:** 10.1186/s13048-022-01058-1

**Published:** 2022-12-03

**Authors:** Zhaowei Cai, Shaojuan He, Rongju Liu, Liling Zhou, Li Zhao

**Affiliations:** 1 Reproductive Center, SSL central hospital of Dongguan, 1 Xianglong Road, Shilong, Dongguan, 523326 Guangdong China; 2 Department of clinical laboratory affiliated Dongguan hospital (Dongguan People’s Hospital), Southern Medical University, Dongguan, 523059 Guangdong China

## Abstract

The survival of ovary granulosa cells (GC) is critical in the initiation and progression of polycystic ovary syndrome (PCOS) in females. Here, we found that the PCOS process is accompanied by massive GC pyroptosis resulting from Caspase-1 inflammasome activation. Administration of plumbagin, an effective compound isolated from plant medicine, can prevent the pyroptosis of GC and the onset of PCOS. Mechanistic study indicates the over-activation of the inflammasome in GC is due to the upregulation of WTAP, a key regulator of the RNA N6-methylase complex. WTAP mediates the mRNA N6-methylation of NLRP3 inflammasome component ASC and enhances ASC RNA stability, which results in the overactivation of the inflammasome in GCs from the PCOS model. Plumbagin treatment suppresses the WTAP-mediated N6-methylation of ASC mRNA and reduces the pyroptosis of GCs. This study supports the profound potential of plumbagin in PCOS treatment.

**Graphical Abstract:**

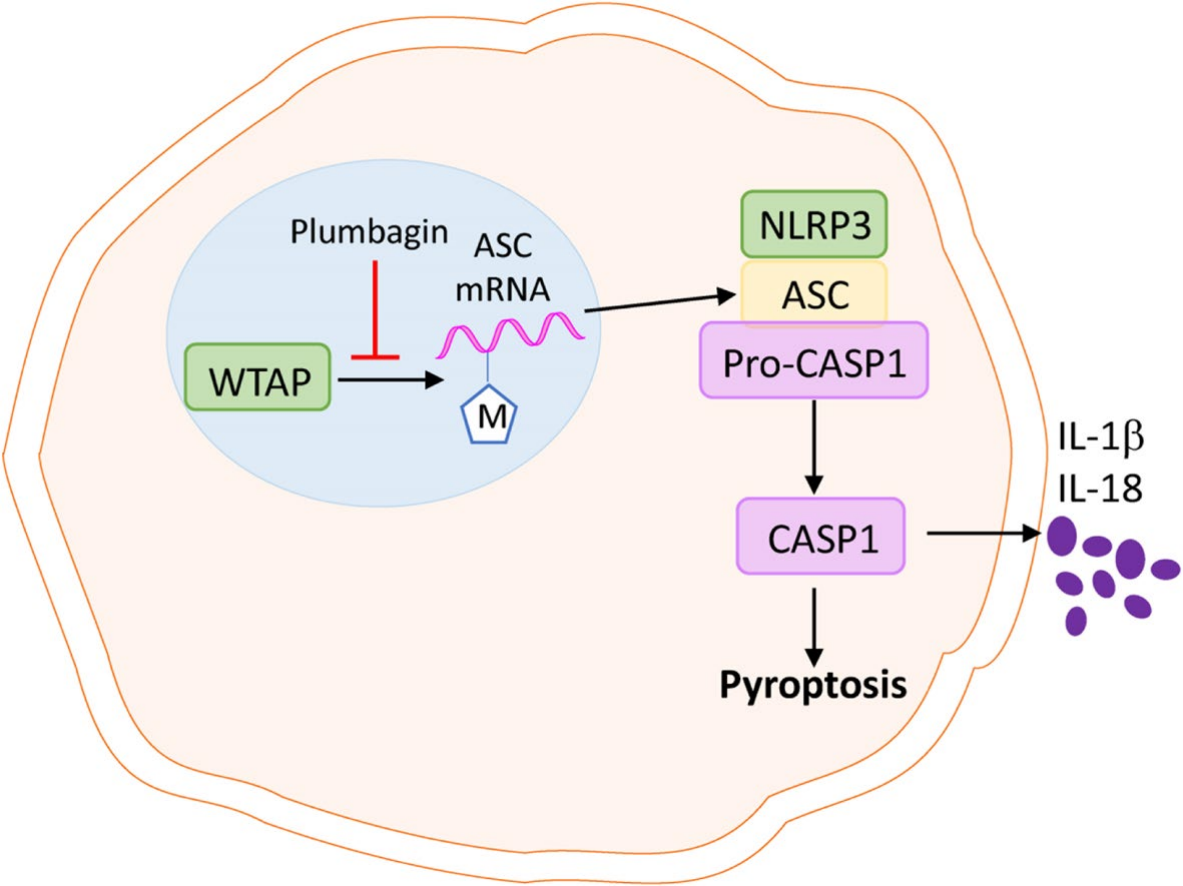

**Supplementary Information:**

The online version contains supplementary material available at 10.1186/s13048-022-01058-1.

## Introduction

Polycystic ovary syndrome (PCOS) is the most common endocrine disorder affecting women of reproductive age, the prevalence of PCOS was between 5 and 15% [1, 2]. The clinical diagnosis and symptoms of PCOS mainly include chronic anovulation and hyperandrogenism [3]. In addition, PCOS is also considered to be closely related to symptoms such as hirsutism, acne, obesity, and insulin resistance [4, 5]. In PCOS women, despite the high number of follicles, the amount of high-quality matured oocytes is very limited, which massively suppresses their intrinsic ability of meiotic maturation and fertilization [6]. Folliculogenesis is the process of ovarian follicle development from primordial germ cells to mature oocytes. During this process, the most abundant cell type in the ovarian, granulosa cell (GC) behavior plays a significant role in supporting oocyte development and producing reproductive hormones such as estrogen, progesterone and other hormones [7, 8]. The cause of PCOS remains largely unknown, but studies suggest the abnormal of GC activity may be closely related to the pathogenesis of PCOS. Studies have shown that apoptosis of GC increased in PCOS which induces the premature atresia of follicular [9, 10]. A previous study has shown that compared with women who have regular ovulatory cycles, a lower level of GC Caspase-3-dependent apoptosis was found in PCOS patients [11]. This suggests the GC cell cycle is closely related to the onset of PCOS, but how GC fits in the progress of PCOS remains not well understood.

PCOS patients were shown to have a higher level of chronic inflammation. A clinical study demonstrated that patients with PCOS have significantly higher levels of C-reactive protein compared with age-matched control individuals [12]. Other inflammation signals such as inflammatory cytokines and oxidative stress were also highly expressed in PCOS patients [13]. This suggests a mechanism in PCOS other than the GC apoptosis, since multiple studies have shown apoptosis does not trigger pro-inflammatory reactions [14–16]. On the other hand, inflammatory status is indeed highly relevant to some other programmed cell death pathways, particularly pyroptosis [17, 18]. Thus, it will be worthwhile to evaluate the pyroptotic-related signal in PCOS. Pyroptosis is an inflammatory programmed cell death pathway activated by human and mouse caspase-1 inflammasome which was triggered by external pathogens or endogenous danger signals, pattern recognition receptors will sense and activate the NOD-like receptors containing pyrin domains (NLRPs) composed of inflammasomes [19]. NLRP3 is a tripartite protein that contains a pyrin domain. The pyrin domain of NLRP3 interacts with the pyrin domain of the apoptosis-associated speck-like protein containing a caspase-recruitment domain (ASC) to initiate inflammasome assembly [20]. The assembly of NLRP3 inflammasome results in the cleavage of pro-Caspase 1 and the release of cytokine IL-1β and IL-18 by cells [21]. Activated caspase-1 cleaves gasdermin D (GSDMD), which causes the N-terminal domain of GSDMD to form pores in the plasma membrane and cause pro-inflammatory cell death, pyroptosis [22, 23]. Given PCOS patients are under higher inflammatory status, the pyroptotic cell death pathway is very likely to be overactivated in ovary cells such as GCs. The detailed regulation pattern of how GC pyroptosis involves in the onset and progression of PCOS needs further investigation.

Plumbagin (PLB; 5-hydroxy-2-methyl-1,4-naphthoquinone) is the effective compound of the medicinal plant Plumbago zeylanica L [24]. In the past few decades, studies suggest plumbagin may play a role in the treatment of multiple types of cancers [25, 26]. Plumbagin was shown to be able to inactivate the major pathways involved in cancer cell expansion including Akt/NF-kB, MMP-9, and VEGF pathways, which will prevent the development of cancer [26]. In experimental rat models, administration of plumbagin was found to prevent the pathogenesis of PCOS, this is due to plumbagin being able to rescue the pathologic low apoptotic rate of GCs in PCOS [27]. Further, plumbagin was proven to have anti-inflammatory effects to ameliorate inflammation diseases such as rheumatoid arthritis and sepsis [28–30]. This suggests plumbagin may also play a role in suppressing the inflammation-related signals to attenuate the development of PCOS pathology.

In this study, we revealed that during PCOS progression, massive GC pyroptosis occurs due to the over-activation of the Caspase-1 inflammasome. Plumbagin treatment can effectively reduce the pyroptosis of GCs. Further studies showed that overexpression of RNA N6-methylase complex member WTAP in GC methylates stabilizes the mRNA of inflammasome component ASC, thereby inducing GC pyroptosis in PCOS. The administration of plumbagin prevents WTAP from methylating ASC which alleviates GC pyroptosis and PCOS development.

## Results

### Plumbagin attenuates the pathological damage and granular cell death in PCOS mice

**Fig. 1:**
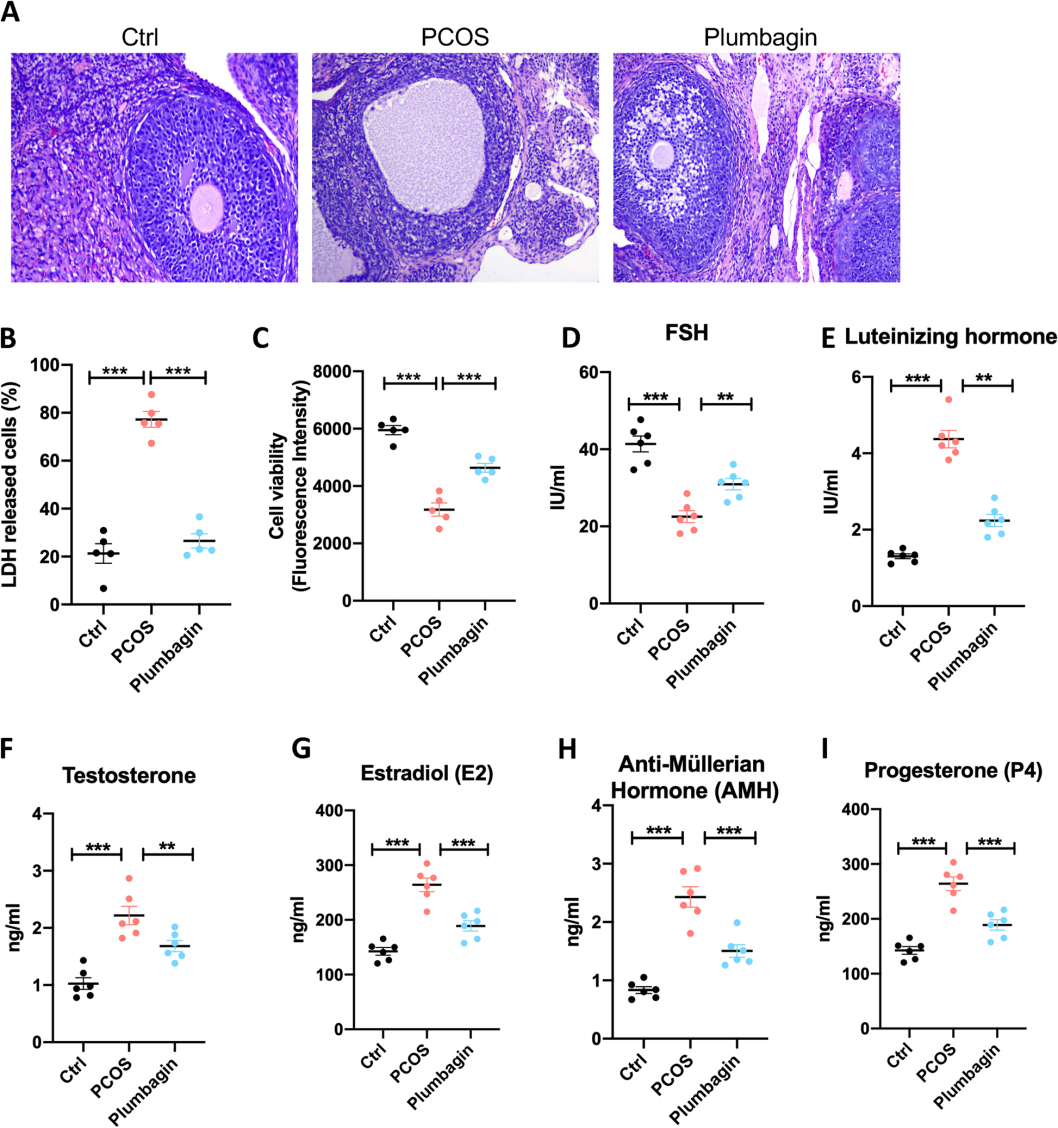
Plumbagin attenuates PCOS in mice by preventing granulosa cell death. **A** H&E staining for the ovary structure. The study includes control mice, PCOS mice and PCOS mice treated with Plumbagin. **B**, **C** GCs were purified from the ovary of three groups of mice, cell survival was determined by three different approaches (*n* = 5). B. LDH release by ovarian GCs in the culture supernatant. **C **Relative cell viability index determined by the fluorescent intensity of alamarBlue™ Cell Viability Reagent staining. **D**-**I** Plasma was isolated from mice in three study groups, reproduction hormone levels were measured by ELISA (*n* = 6). D. Follicle-Stimulating Hormone (FSH). **E** Luteinizing Hormone (LH). **F** Testosterone. **G** Estradiol(E2). **H** Anti-Müllerian Hormone (AMH). **I** Progesterone (P4). Individual data points are displayed. Data are mean ± SEM. Unpaired one-way ANOVA was used to analyze the difference. *P < 0.05; **P < 0.01; ***P < 0.001

To evaluate the role of Plumbagin in treating PCOS, we created a mice model for PCOS [31, 32]. On day 20 of PCOS induction, a disruption of the mice’s ovarian was observed by H&E staining (Fig. 1A). Compare with the control group, PCOS mice displayed increased cystic follicles and hyperemia. The thickness and arrangement of the granulosa cell (GC) layer were heavily destroyed (Fig. 1A). When the mice were treated with plumbagin for 3 weeks, the pathogenesis in the ovarian and the GC layer was significantly reversed (Fig. 1A). To further determine the disturbance of the GCs in the PCOS model, primary GCs were isolated and cultured *ex vivo*. The survival rate of the GCs was measured with two different approaches: quantification of lactate dehydrogenase (LDH) release by dead cells and staining with alamarBlue™ cell viability reagent [33]. Strikingly, we found about 80% of GCs in PCOS mice were prone to release LDH in culture, while only about 20% GCs in the control group underwent cell death (Fig. 1B). Administration of plumbagin reduced the death rate of PCOS GCs to about 25%, which is very close to the normal range (Fig. 1B). Similarly, in the cell viability test, GCs from PCOS mice display reduced cell viability, while plumbagin treatment remarkably improved the GC survivability (Fig. 1C).

To check the ovary function in detail, a group of hormones was measured in the plasma of each study group. A typical FSH^lo^ LH^hi^ phenotype was found in the PCOS mice model which was reduced by the injection of plumbagin (Fig. 1D, E). Other hormones including testosterone, E2, AMH and P4 were also increased in the PCOS model, plumbagin administration effectively reversed the levels of those hormones (Fig. 1F-I). Together, we found plumbagin treatment impressively suppresses the pathogenesis and GC cell death in the PCOS mice model.

### Plumbagin lowers the heightened GC pyroptosis in PCOS

**Fig. 2:**
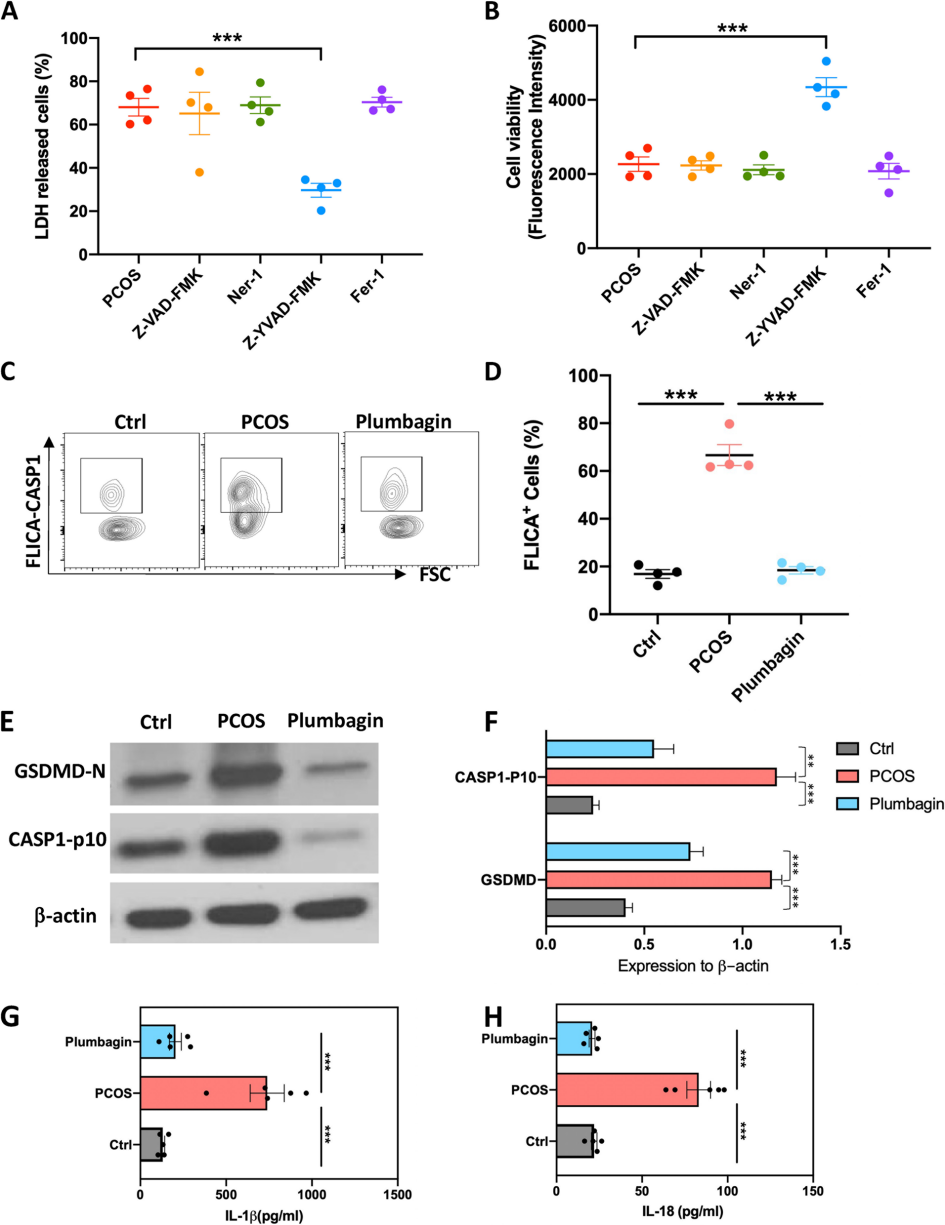
Plumbagin reduced the inflammasome overactivation and pyroptotic death of Granulosa cells in PCOS mice. **A**, **B** GCs from PCOS mice were isolated and cultured for 48 hours. Inhibitors for different cell death pathways were added as labeled in the figure. Cell survival was quantified with two approaches. *n* = 4. A. LDH release to the culture medium. **B** Relative cell viability by alamarBlue™ Cell Viability Reagent staining. **C**-**F** GCs were isolated from the ovary of control, PCOS and PCOS mice treated with Plumbagin, the activation of inflammasome was measured. **C** Activation of Caspase-1 indicated by staining with FLICA probe (Representative graph). **D** Activation of Caspase-1 indicated by staining with FLICA probe (statistic, *n* = 4). E. Immunoblot for the amount of active fragment Caspase-1-p10 and GSDMD-N. F. Protein level of Caspase-1-p10 and GSDMD-N normalized with b-actin. *n* = 3. G. IL-1β level in the culture supernatant were detected by ELISA after 24 hours. *n* = 5. H. IL-18 release to the culture medium after 24 hours were quantified with ELISA. *n* = 5. Individual data points are displayed. Data are mean ± SEM. Unpaired one-way ANOVA were used to analysis the difference. *P < 0.05; **P < 0.01; ***P < 0.001

To investigate how GC dead in the PCOS mice, we performed a screening of cell death pathways with GCs from PCOS mice. Generally, GCs were treated with inhibitors of different cell death pathways including apoptosis (Z-VAD-FMK), narcosis (Ner-1), pyroptosis (Z-YVAD-FMK) and ferroptosis (Fer-1). Pyroptosis inhibitors successfully reduced the amount of LDH-released GCs, while other inhibitors did not show an impressive effect (Fig. 2A). The overall cell viability was also increased after inhibition of pyroptosis, blockade of other death routes did not make a difference in GC survival (Fig. 2B).

Pyroptosis was initiated by the active cleavage of inflammasome Caspase-1, we then tested the activation of Caspase-1 with a specific FLICA probe [34]. As a result, cleavage of Caspase 1 was detected in over 60% of GCs from the PCOS mice, while control mice only display about 20% of Caspase-1 activation in GCs (Fig. 2C, D). Plumbagin treatment brings the GC caspase-1 cleavage back to normal level (Fig. 2C, D). Inflammasome activation gives rise to the formation of Gasdermin pores on the cell membrane, which can be monitored by the cleavage of protein Gasdermin D (GSDMD) [35]. We then use immunoblot to test the cleavage of Caspase 1 and GSDM. As shown in Fig. 2E, F**,** compared with the control, an enhanced signal of Caspase-1 p10 and GSDMD was detected in GCs from PCOS mice. Plumbagin reduced the Caspase-1 p10 and GSDMD level back to normal (Fig. 2E, F). Pyroptosis was also accompanied by the release of cytokines IL-1β and IL-18, we also measured the accumulation of these two cytokines in the culture supernatant of GC cells. GCs from PCOS mice released more IL-1β (Fig. 2G) and IL-18 (Fig. 2H) than control mice, administration of Plumbagin again rescued the cells from producing those cytokines (Fig. 2G, H). These data indicate the Plumbagin treatment can attenuate the increased pyroptosis of GCs in PCOS mice.

### Inhibition of inflammasome activation rescues the pathogenesis of PCOS

**Fig. 3:**
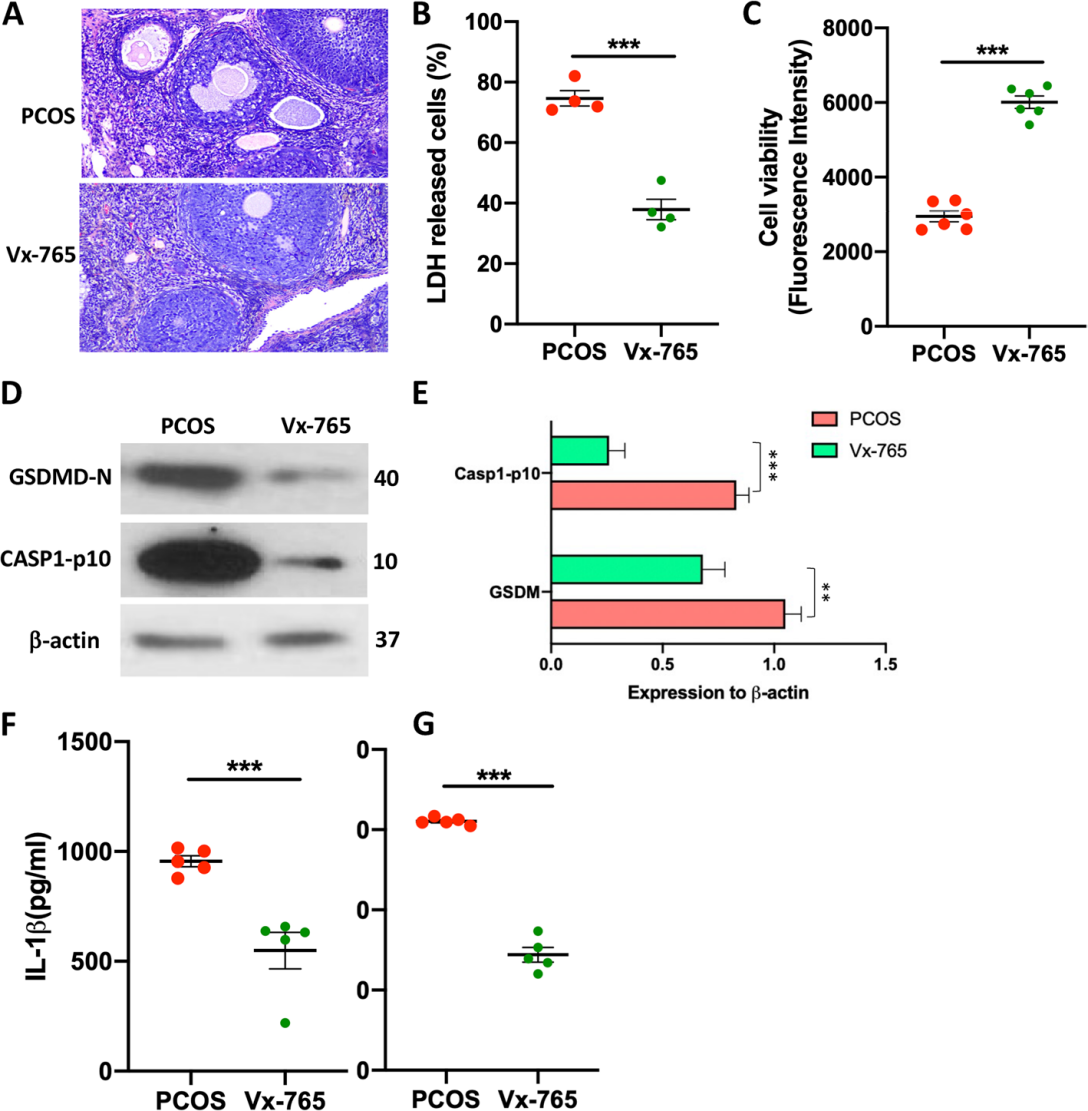
Caspase 1 inhibitor Vx-765 rescues the PCOS pathology by preventing the pyroptosis of GCs. PCOS mice were treated with Caspase 1 inhibitor Vx-765. **A** H&E staining for the ovary structure. **B** The LDH released by GC cells. *N* = 4. **C** GC viability was measured by alamarBlue™ Cell Viability Reagent staining. *N* = 6. **D** Immunoblot to quality active fragment Caspase-1-p10 and GSDMD-N. **E** Relative protein expression of Caspase-1-p10 and GSDMD-N normalized with b-actin. *N* = 3. **G** IL-1β concentration in the culture supernatant was measured by ELISA after 24 hours. **H** The amount of IL-18 released to the culture medium after 24 hours. *N* = 5. Individual data points are displayed. Data are mean ± SEM. Unpaired student t-test were used to analysis the difference. *P < 0.05; **P < 0.01; ***P < 0.001

To investigate whether the activation of the Caspase-1 inflammasome in GCs regulates the development and progression of PCOS in mice, we injected the inhibitor of Caspase-1 Vx-765 into our PCOS mice model. Based on the ovary section H&E staining, we found administration of Vx-765 significantly reduces the development of PCOS (Fig. 3A). We then purified the GCs from the mice model and tested their survival situation. As a result, the Vx-765 treatment effectively reduced the LDH-released dead GCs in the PCOS model (Fig. 3B). Similarly, the blockade of caspase-1 activation enhanced the viability of GCs in PCOS mice (Fig. 3C). Further, immunoblot data suggest that Vx-765 injection reduced the cleavage level of GSDMD and Caspase-1 in the protein level (Fig. 3D, E). The production of IL-1β and IL-18 also decreased along with Vx-765 treatment (Fig. 3F, G).

### Plumbagin treatment suppresses the activation of Caspase-1 in Granulosa cells

**Fig. 4:**
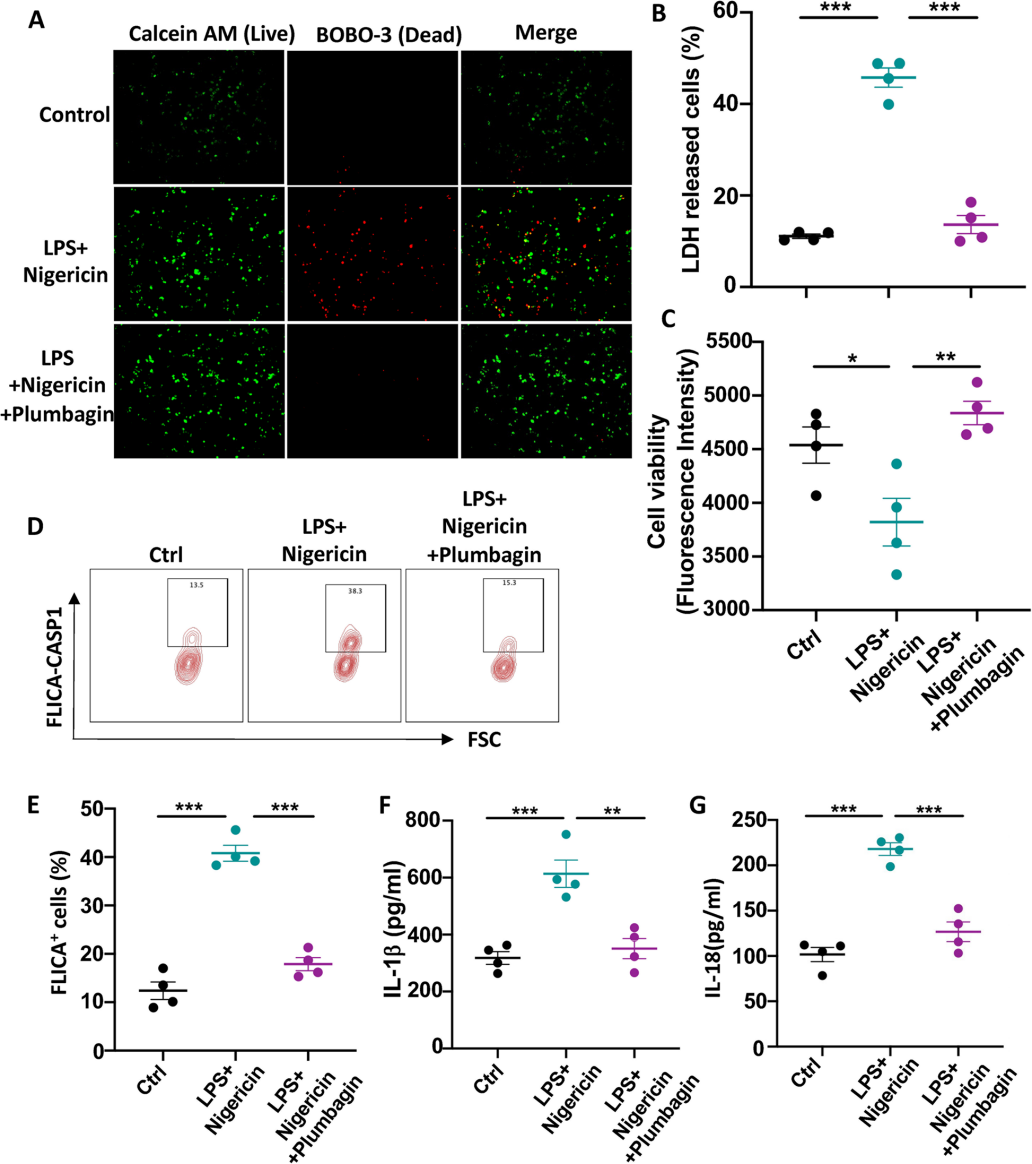
Plumbagin protects against the pyroptotic death of Granulosa cells. GC cell line HGL5 was cultured in presence of pyroptosis inducer nigericin and plumbagin. **A** Cell survival was determined by LIVE/DEAD™ Cell Imaging. **B** The production of LDH by dead cells was quantified. *N* = 4. **C** The cell viability of HGL5 was measured by alamarBlue staining. *N* = 4. **D** Activation of Caspase-1 indicated by staining with FLICA probe (Representative graph). **E** Activation of Caspase-1 indicated by staining with FLICA probe (statistic). *N* = 4. **F** IL-1β concentration in the culture supernatant was measured by ELISA. *N* = 4. G IL-18 release to the culture medium was quantified by ELISA. *N* = 4. Individual data points are displayed. Data are mean ± SEM. Paired one-way ANOVA was used to analyze the difference. *P < 0.05; **P < 0.01; ***P < 0.001

To further confirm the role of Plumbagin specifically in protecting granulosa cells, we induced pyroptosis of the human GC cell line HGL5 in vitro with a combination of LPS and nigericin [36]. With a cell live/death staining approach, we found that LPS and nigericin induced a massive amount of GC death after overnight culture (Fig. 4A). Adding plumbagin in culture directly reduced the death of GCs under pyroptosis induction (Fig. 4A). Overnight LPS and nigericin treatment-induced about 50% GCs death which was quantified by the release of LDH, plumbagin rescued the GCs from the LPS, and nigericin-mediated pyroptosis (Fig. 4B). The GC survival was also measured with a cell viability probe, which again showed plumbagin plays a protective role in the GCs (Fig. 4C).

We detected the Caspase 1 cleavage with the FLICA probe to investigate how plumbagin regulates inflammasome activation. Pyroptosis inducer, LPS, and nigericin enhanced the active cleavage of caspase 1 which was blocked by co-culture with plumbagin (Fig. 4D, E). Pyroptosis was also quantified with the release of IL-1β and IL-18, we found that LPS and nigericin treatment results in robust production of IL-1β and IL-18 while plumbagin reversed the release of those pyroptotic cytokines (Fig. 4F, G). These data with GC cell line demonstrated plumbagin prevents the GCs from undergoing Caspase-1 mediated pyroptosis.

### Plumbagin destabilizes the mRNA of the inflammasome compartment ASC

**Fig. 5:**
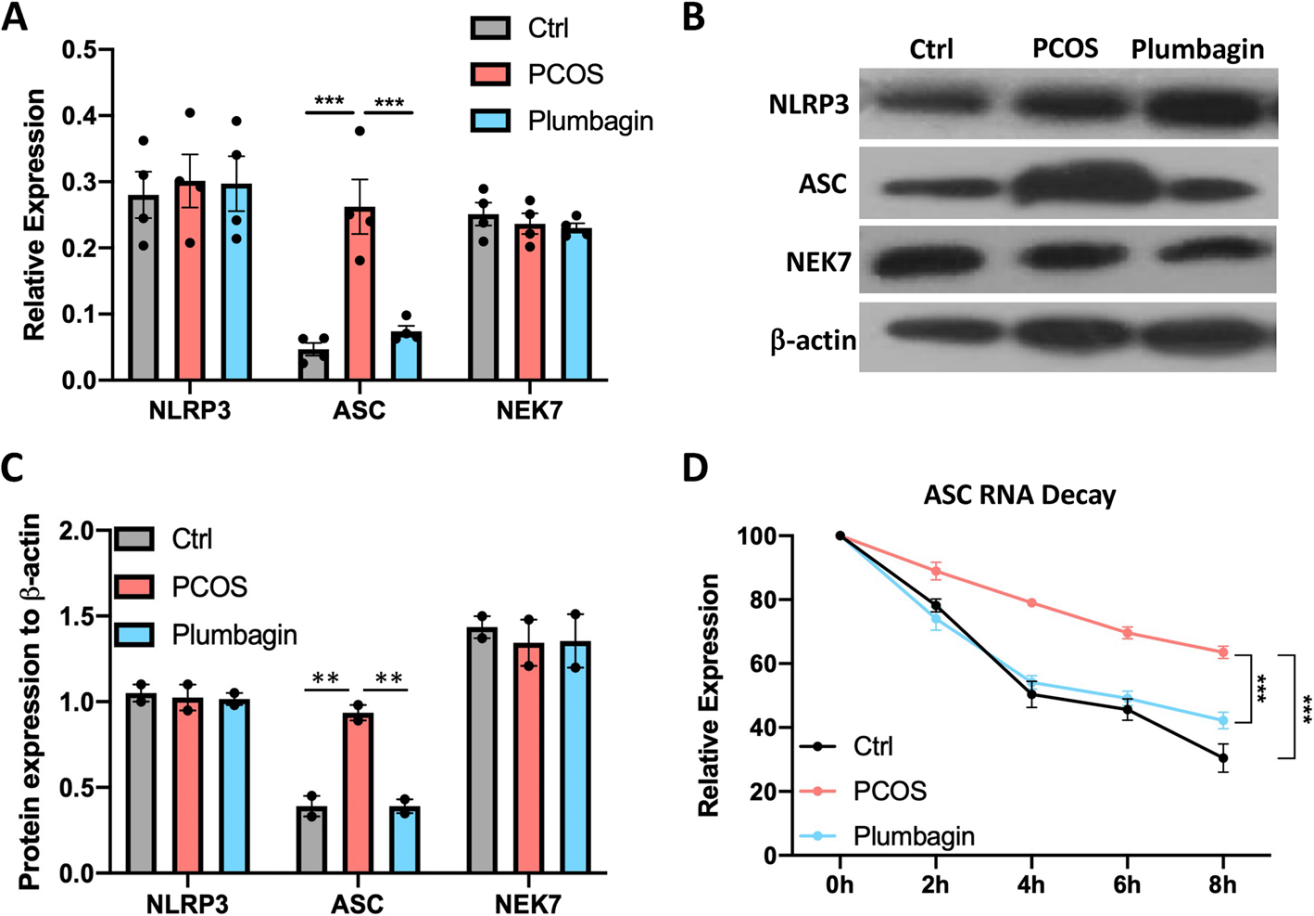
Plumbagin destabilizes the mRNA of the inflammasome component ASC. GCs were isolated from the ovary of mice. **A** The mRNA relative expression of inflammasome complex NLRP3,ASC and NEK7 were measured by RT-PCR. *N* = 4. **B** Protein accumulation of NLRP3,ASC and NEK7 were detected with immunoblot. **C** Relative protein expression of NLRP3,ASC and NEK7 normalized to b-actin. *N* = 3. **D** GCs were treated with transcription inhibitor actinomycin D, and the decay of ASC mRNA was measured every 2 hours for 8 hours. Individual data points are displayed in the bar graph. Data are mean ± SEM. one-way ANOVA was used to analyze the difference in **A** and **C**. Two-way ANOVA was used to analyze the difference in **D**. *P < 0.05; **P < 0.01; ***P < 0.001

The activation of NLRP3 inflammasome is in response to the endogenous danger signals [37, 38]. Inflammasome activation requires the assembly of the NLRP3 and the adaptor protein ASC, recent studies also indicate protein NEK7 is essential for NLRP3 inflammasome activation. The assembly of the NLRP3 complex results in the cleavage and activation of Caspase 1 and the release of cytokines IL-1β and IL-18 [39]. To understand how plumbagin regulates the activation of the inflammasome, we took the GCs from control, PCOS, and plumbagin-treated PCOS mice, the expression of NLRP3 inflammasome key compartments was quantified. At the transcriptional level, we found a massive accumulation of ASC mRNA in GCs from PCOS mice which was reduced by plumbagin treatment (Fig. 5A). Meanwhile, the transcripts of NLRP3 and NEK7 were not changed in PCOS or plumbagin treatment. Immunoblot was used to determine the protein expression of the NLRP3 complex, again, an increase of ASC protein was observed in the GCs from PCOS mice. The administration of plumbagin adjusted ASC protein back to normal level (Fig. 5B, C). The protein level of NLRP3 and NEK7 were unchanged in the control and PCOS groups (Fig. 5B, C).

The accumulation of gene transcripts depends on the transcription level and stability of the mRNA [40]. To figure out whether the upregulated ASC mRNA is due to increased transcription or enhanced RNA stability, we performed an ASC RNA decay assay with a transcription inhibitor actinomycin D. Interestingly, we found the decay of ASC mRNA reduced and slowed down in GCs from PCOS mice, plumbagin treated mice display similar dynamic in ASC mRNA decay compare with the control group (Fig. 5D). Taken together, these data indicate PCOS is associated with enhanced stability of ASC mRNA in GCs, which in turn causes the overactivation of caspase-1 inflammasome. Plumbagin treatment destabilized ASC mRNA and prevent inflammasome activation in GCs.

### Overexpression of WTAP in PCOS GCs gives rise to the N-6 methylation and stabilization of ASC mRNA

**Fig. 6:**
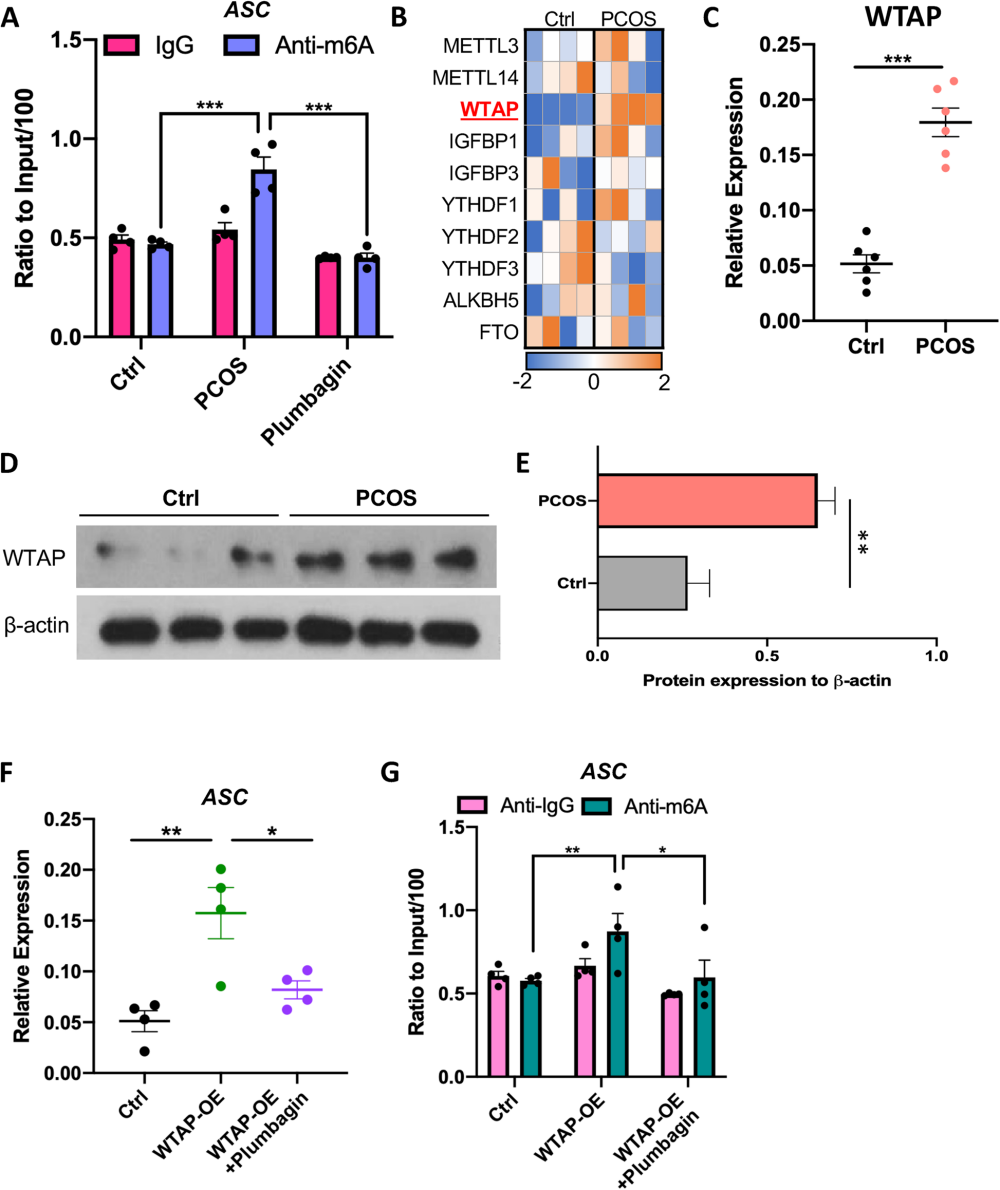
Increased WTAP expression promotes the N6-methylation and stabilization of ASC mRNA in GCs from PCOS mice. **A** Methylated RNA Immunoprecipitation (Me-RIP) assay to quantify m6A level of ASC mRNA in GCs from control, PCOS and Plumbagin-treated mice. *N* = 4. **B** Relative expression of m6A-related genes in GCs from three experimental mice groups. *n* = 4. **C** WTAP transcripts in the GCs of control and PCOS mice were quantified by RT-PCR. **D** WTAP protein in control and GCs were detected by immunoblot. **E** Relative WTAP protein level normalized with β-actin. *N* = 3. **F** Overexpress WTAP in GC cell line HGL5 by transfecting with a recombinant plasmid containing WTAP. The relative expression of ASC mRNA in HGL5 was determined by RT-PCR. *n* = 4. **G** Relative m6A level of ASC mRNA level in HGL5 cells were enriched and quantified by Me-RIP. *n* = 4. Individual data points are displayed. Data are mean ± SEM. one-way ANOVA were used to analysis the difference in A,F,G; student t-test was used to analysis difference in C and E. *P < 0.05; **P < 0.01; ***P < 0.001

The modifications of RNA were proved to be associated with the stability of mRNA. Of which, N6-Methyladenosine (M6A) was the most abundant type of mRNA modification [41, 42]. M6A regulates mRNA amount by many means including pre-mRNA processing, nuclear export, decay, and translation. Ultimately, the target mRNA will stabilize or decay or undergo alternative splicing. Since we see the decay of ASC mRNA in PCOS mice, we wonder whether the ASC mRNA was being methylated. To determine the m6A level of ASC in GCs from PCOS mice, we use an m6A-specific antibody to pull down the mRNA in GCs from control, PCOS and plumbagin-treated mice, IgG antibody was used as the internal control. As a result, the m6A degradation of ASC mRNA was significantly higher in the GCs of PCOS mice compared with the control (Fig. 6A). As predicted, the m6A level of ASC mRNA was reduced by plumbagin treatment (Fig. 6A). A bunch of molecular and enzymes is involved in the process of m6A including the ‘writer’ methyltransferase, the “eraser” demethylases, and the ‘reader’ proteins that determine the mRNA fate after being methylated [43]. Therefore, we tested the expression of m6A-related genes in the GCs of control and PCOS mice. After screening the transcription of 10 m6A key enzymes, we found the expression of the m6A ‘writer’ methyltransferase complex component WTAP significantly increased in the GCs of PCOS mice (Fig. 6B, C). We then confirmed the protein expression of WTAP in GCs with immunoblot, again, we found an increase of WTAP protein in the GCs of PCOS mice compared with controls (Fig. 6D, E).

To test whether the overexpression of WTAP is responsible for the elevation of ASC, we overexpressed WTAP in the human GC cell line HGL5. Overexpression of WTAP in HGL5 with plasmid transfection increased the expression of ASC transcripts, while treated WTAP overexpressed HGL5 with plumbagin reversed ASC mRNA expression back to normal (Fig. 6F). We also performed RNA immunoprecipitation with an anti-m6A antibody, overexpression of WTAP increased the m6A of ASC in HGL5 cells which were suppressed by plumbagin treatment (Fig. 6G). Together, we found an elevation of key m6A enzyme WTAP in PCOS, which is associated with the enhanced m6A and stability of ASC mRNA in PCOS GCs. Treatment with plumbagin reduced the m6A of ASC mRNA, which in turn suppressed the expression of ASC.

### Plumbagin retards the WTAP-ASC axis-mediated pyroptosis in granulosa cell

**Fig. 7:**
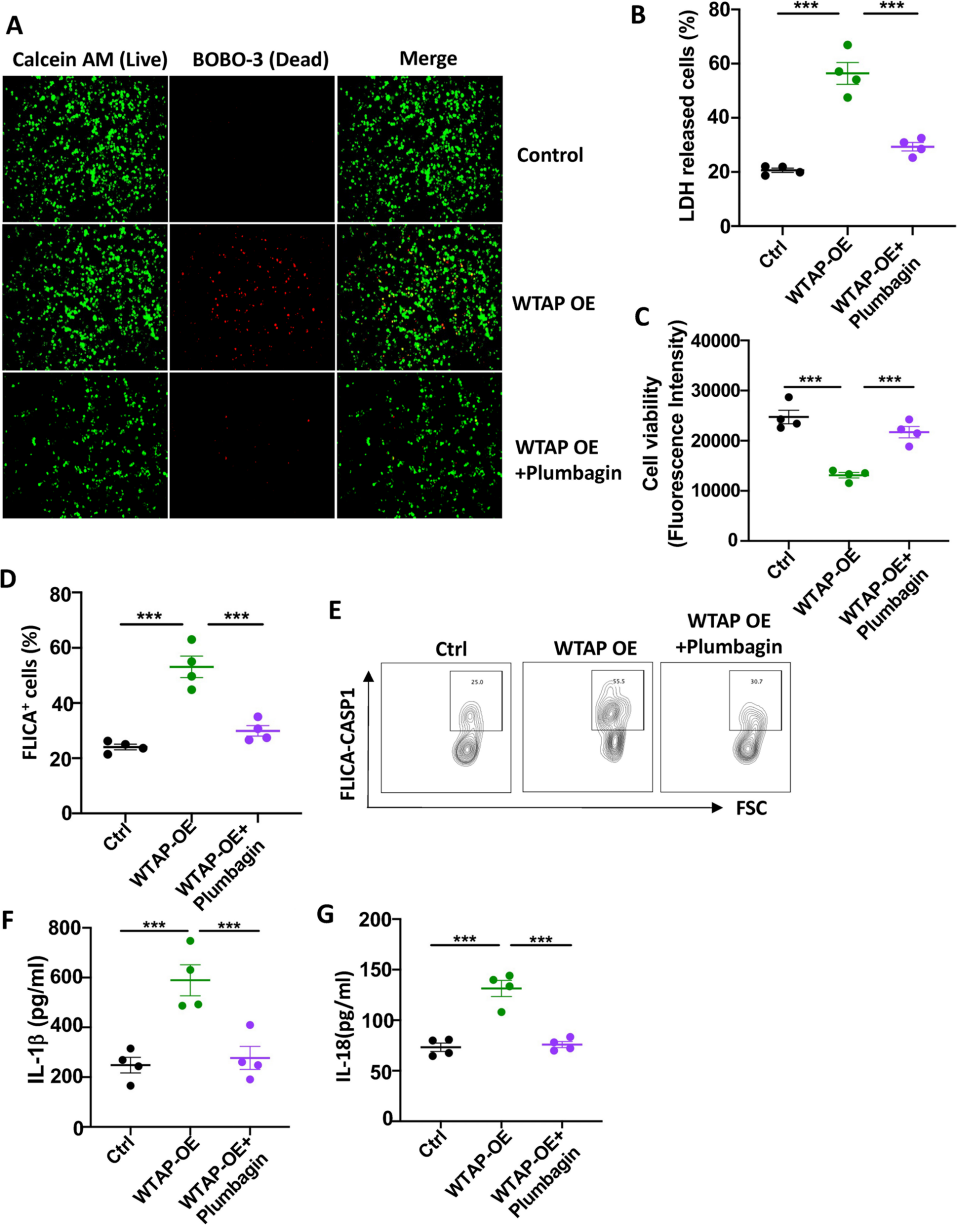
Plumbagin retards the WTAP-mediated granulosa cell pyroptosis. Overexpress WTAP in GC cell line HGL5 by transfecting with a recombinant plasmid containing WTAP. The cells were then treated with plumbagin. **A** Cell survival imaged by LIVE/DEAD™ Cell Imaging kit. **B** LDH production by dead cells was quantified by LDH kits. *N* = 4. **C** Cell viability was tested with alamarBlue staining. *n* = 4. **D** FLICA probe stain for activated Caspase-1 (Representative graph). **E** FLICA probe stain for activated Caspase-1(statistic).*n* = 4. **F** IL-1β production by HGL-5 cells were quantified by ELISA. *N* = 4. G IL-18 production by HGL 5 cells were determined by ELISA. *N* = 4. Individual data points are displayed. Data are mean ± SEM. Paired one-way ANOVA was used to analyze the difference. *P < 0.05; **P < 0.01; ***P < 0.001

To verify the expression of WTAP regulates the pyroptosis of GCs, we overexpressed WTAP in HGL5 and tested cell survival with three independent approaches. We found overexpression of WTAP increased the death rate and LDH release by GCs, which was reversed by the treatment of plumbagin (Fig. 7A, B). Cell viability dye staining also indicated reduced cell viability when WTAP expression is high, plumbagin again attenuated the cell survival level in WTAP overexpressed HGL5 cells (Fig. 7C).

We also detected overexpression of WTAP and the activation of Caspase-1 inflammasome. FLICA probe staining indicated a higher level of caspase-1 cleavage in WTAP overexpressed HGL5 cells, plumbagin treatment reversed the active cleavage of caspase-1(Fig. 7D, E). A growth of cytokine IL-1β and IL-18 production was detected in WTAP overexpressed GCs, which was again reduced by plumbagin treatment (Fig. 7F, G). Taken together, the increase of WTAP expression results in the elevation of Caspase-1 induced pyroptosis in GCs, treatment with plumbagin effectively prevents the GCs from pyroptosis.

## Methods

### PCOS mice model

Female C57BL/6 mice at 25-day-old were purchased from Guangdong Medical laboratory animal center, Guangdong, China. The mice were maintained under a 12 h light:12 h darkness light cycle with free access to food and water. Animal experimentation was approved by the Animal Ethics Committee of Guangzhou University of Chinese Medicine according to the national legislation for animal care. To generate the PCOS model, mice were subcutaneously injected with dehydroisoandrosterone (60 mg/kg body weight, LKT Laboratories) daily for 20 days [31]. The control group was injected with the same volume of corn oil and 0.01 ml of 95% ethanol daily. For plumbagin treatment, 10 mg/kg of plumbagin was injected into mice intraperitoneally.

### Cell culture

Immortalized Human Granulosa Cells (HGL5) were purchased from Applied Biological Materials Inc. (T0650). The cell was maintained in RPMI-1640 medium supply with 10% Fetal Bovine Serum (FBS) and 1% of Penicillin/Streptomycin Solution. The cell culture medium was changed every other day to maintain cell viability. 10 μM of plumbagin was added to the culture medium in some groups.

### Granulosa cell isolation

Ovaries were removed from mice and punctured with a 25-gauge needle to isolate granulosa cells. Granulosa cell/oocyte mixture was obtained by passing the tissue lysate through a 70 μm cell strainer (BD Falcon, USA). To further purify granulosa cells, the granulosa cell/oocyte mixture was then filtered through a 40 μm cell strainer (BD Falcon, MA, USA), which will separate granulosa cells from oocytes. The purity of granulosa cells was determined by staining isolated cells with an anti-FSHR antibody (Thermo Fisher CL594-22665).

### Tissue harvest and H&E staining

The ovary was harvested from mice and embedded with an optimal cutting temperature compound (OCT, Thermo Fisher 23-730-571) on dry ice. Tissue sections were cut at a thickness of 5 μm. Hematoxylin & Eosin Stain (H&E) staining was performed with the Beyotime staining kit following the manufacturer’s instructions (Beyotime, C0105S, China).

### Hormone measurement

500-600 μl of whole blood was collected from the experimental mice into EDTA pretreated tubes. The whole blood was centrifuged at 800xg for 10 min, plasma was collected from the upper layer. Hormones related to ovary functions were measured by ELISA with the following kits; Mouse Follicle Stimulating Hormone (FSH) ELISA Kit (Abclonal, RK04237), Mouse Luteinizing Hormone (LH) ELISA Kit (Abclonal, RK01779), Mouse Testosterone ELISA Kit (Crystalchem,80,552), Mouse Estradiol ELISA Kit (Biorbyt,orb340108), Mouse Anti-Mullerian Hormone (AMH) ELISA Kit (Abclonal, RK02588), Mouse Progesterone (P4) ELISA Kit (Crystalchem,80,559). All ELISA assays were performed following the manufacturer’s instructions.

### Cell viability measurement

Three methods were used to determine cell survival. The release of Lactate Dehydrogenase (LDH) into the supernatant was measured by the Pierce LDH Cytotoxicity Assay Kit (Thermo Fisher,88,953). Cell viability was quantified with the AlamarBlue Cell Viability Reagent (Thermo Fisher, DAL1025). Live and dead cells were stained with the LIVE/DEAD™ Cell Imaging Kit (488/570) (Thermo Fisher, R37601). All assays were performed following the manufacturer’s instructions.

### Caspase-1 FLICA staining

FAM-FLICA® Assay Kit for Caspase 1 was purchased from Immunochemistry Technologies. 1:50 of FLICA in PBS were incubated with cells for 1 hour at 4 °C and analyzed with flow cytometer.

### Immunoblot

Protein was isolated by lysis cell pellets in RIPA buffer for 30 mins at 4 °C. The proteins were resolved in 4-15% SDS-PAGE and transferred to PVDF membranes (Bio-Rad, 1,620,177). The membrane was blocked in 1%BSA/TBST for 1 hour at RT. Primary antibodies were diluted in TBST and incubated with the membrane at 4 °C overnight. The membrane was then incubated with HRP-conjugated secondary antibody for 1 hour at RT after 3 washes with TBST. Pierce™ ECL Western Blotting Substrate (Thermo Fisher, 32,132) was used to detect protein.

### ELISA

Culture medium was collected and IL-1β and IL-18 were quantified with ELISA kit (IL-1β: Biolegend 432,604; IL-18: Abcam ab216165) following the manufacturer’s instructions. Generally, the culture medium was added to an antibody pre-coated microplate, then rotated and incubated at room temperature for 2 hours. The plate was washed three times and detection antibody was added to the plate and allowed to incubate for 1 hour. HRP solution was added to the plate, after three washes, for 30 mins. The plate was washed six times, and TMB substrates were added for 15 min. The reaction is stopped using a stop buffer, and the absorbance is measured at 450 nm wavelength.

### RNA isolation and RT-PCR

Total RNA was extracted using Trizol RNA isolation kit (Beyotime, R0011). Reverse transcription was performed with cDNA Synthesis Kits following the manufacturer’s instruction (Thermo Fisher, 4,368,813). Quantitative PCR was performed with SYBR Green qPCR Master Mix (Bimake, B21202). Gene expression was normalized to beta-actin. Sequence of primers used in this study was provided in the supplementary table.

### Me-RIP

Me-RIP assays were performed using EpiQuikTM CUT&RUN m6A RNA Enrichment kits (EpiGentek, P-9018) following the manufacturer’s instructions. In brief, 15 μg of total RNA was incubated with beads bounded m6A capture antibody for 90 minutes at RT. The enriched RNA fragments were eluted and purified with RNA-binding magnetic beads. mRNA was reverse transcribed into cDNA, the amount of ASC mRNA was quantified with qRT–PCR.

### RNA decay assay

mRNA was extracted and treated with 10 μg/ml of transcription inhibitor Actinomycin. Samples were harvested every two hours up to 8 hours. RNA was reverse transcribed to cDNA and quantified with QRT-PCR as described before.

### Statistic

All data was presented as mean ± standard error of the mean (S.E.M), *P* < 0.05 was considered statistically significant. Two-tailed unpaired Student’s t-test and one-way ANOVA were used to compare groups. Statistical analyses was performed with Prism GraphPad 6.0 (GraphPad Software Inc.).

## Discussion

As the most abundant cell type in the ovary, granulosa cells (GC) plays a crucial role in the development and maturation of the follicles. During the folliculogenesis process, the dysregulation of GC lifespan will lead to several reproduction diseases for women, one of the major diseases is PCOS, which causes female infertility in 5-15% of females worldwide. The cause of PCOS was not well understood yet. Previous studies have shown that PCOS is related to long-term inflammatory status and the reduced apoptotic rate of GCs [11, 12, 44]. In this study, using a PCOS mouse model and the human GC cell line HGL5, we attempted to further understand the detailed machinery underlying the GC behavior in PCOS. Plumbagin, the effective compound from plant medicine, attenuated PCOS [27]. Here, we further explored how plumbagin treats PCOS by preventing the pyroptotic cell death of the GCs.

In the ovary of PCOS mice, enhanced activation of Caspase-1 inflammasome was detected in the GCs, resulting in the pyroptosis of GCs. Plumbagin treatment might reverse this process. This uncovered the mystery of the GC fate in the process of PCOS. This funding closely links the function of plumbagin with the regulation of inflammasome activation and cell pyroptosis. Specifically, the expression of the RNA methylation complex component WTAP is upregulated in GCs from PCOS mice, which allows a large amount of m6A modification on the mRNA of ASC, a critical protein in NLRP3 inflammasome. Accumulation of ASC mRNA leads to the overactivation of inflammasome in GCs and the pyroptosis of these cells. The administration of plumbagin plays a role in limiting the WTAP-mediated m6A of ASC and suppressing the pyroptosis of GCs ultimately. Overall, the general process and mechanism of this study was shown in Graphical Absract.

This study updates our understanding of PCOS on many different levels and provides potential new targets for the treatment of PCOS. First, we clarified that in the process of PCOS, the massive damage of GC stems from a specially programmed death mechanism called pyroptosis. This suggests that more research and medicine targeting the pyroptosis pathway is needed for PCOS treatment in the future. Second, previous studies have rarely addressed how the modifications at the mRNA level affect the development of diseases such as PCOS. Here, we show that a specific mRNA modification, m6A, is able to induce the activation of inflammasome leading to GC pyroptosis. Therefore, m6A-specific inhibitors are likely to be used in the treatment of PCOS in the future. Finally, we found that plumbagin, the active ingredient in botanical medicine, could effectively reverse the progress of PCOS, the mechanism was also displayed in this study. This strongly supports the potential of plumbagin to be widely used in the treatment of PCOS. Our current study is based on in vitro cell lines and in vivo mouse models, which limits the extent to which the study can be applied. However, the drug targets and molecular mechanisms shown in this study provide a solid theoretical basis and data support for further clinical and patient-level study in the treatment of PCOS.

## Supplementary Information

**Additional file 1.**
